# Effects of physical training with different intensities of effort on lipid metabolism in rats submitted to the neonatal application of alloxan

**DOI:** 10.1186/1476-511X-11-138

**Published:** 2012-10-15

**Authors:** Carla Ribeiro, Lucieli Teresa Cambri, Rodrigo Augusto Dalia, Michel Barbosa de Araújo, José Diego Botezelli, Amanda Christine da Silva Sponton, Maria Alice Rostom de Mello

**Affiliations:** 1 Institute of Biosciences Physical Education Department, São Paulo State University – (Universidade Estadual Paulista- UNESP), Av: 24-A, 1515 Bela Vista, Rio Claro – São Paulo, CEP: 13506-900, Brazil

**Keywords:** Neonatal rats, Alloxan, Exercise training, Lipid metabolism

## Abstract

**Background:**

Type 2 diabetes mellitus (T2DM) is a chronic disease that is characterized by insulin resistance. Its development is directly connected with the inability of insulin to exert its action, not just on carbohydrate metabolism but also on primarily on lipid metabolism. The present study aimed to compare the effects of continuous, intermittent, and strength training on serum and tissue variables on the lipid metabolism of alloxan rats.

**Methods:**

Wistar rats were divided into eight groups: sedentary alloxan (SA), sedentary control (SC), continuous training alloxan (CA), intermittent training alloxan (IA), strength training alloxan (StA), continuous training control (CC), intermittent training control (IC) and strength training control (StC). Alloxan (250 mg/kg bw) was injected into neonatal rats at 6 days of age. The continuous training protocol consisted of 12 weeks of swimming training for 1 uninterrupted hour / day, five days/ week, supporting a load that was 5% bw. The intermittent training protocol consisted of 12 weeks of swimming training with 30 s of activity interrupted by 30 s of rest, for a total of 20 min/day, five days/ week, supporting a load that was 15% bw. The strength-training protocol consisted of 12 weeks of training, five days/week with 4 sets of 10 jumps in water with 1 min rest between sets, supporting a load that was a 50% bw.

**Results:**

At 28 days, the alloxan animals exhibited higher insulin resistance as measured by the disappearance of glucose serum (% Kitt/min) during the ITT. At 120 days, the sedentary alloxan animals showed higher FFA values than continuous and intermittent training alloxan. In addition, the alloxan animals that underwent intermittent and strength training showed lower FFA values compared to the corresponding controls. The continuous training protocol was less effective than the strength training protocol for reducing the levels of total cholesterol in the alloxan animals. Serum total lipid values revealed that intermittent training increased serum levels in alloxan animals

**Conclusion:**

Thus, it was concluded that physical training at different intensities of effort is of great importance in attenuation and control of changes in the lipid metabolism in alloxan animals.

## Introduction

In recent years, the incidence of type 2 diabetes mellitus (T2DM) has increased considerably [1]. T2MD is a chronic disease that is characterized by insulin resistance [2,3] and Its development is directly associated with the inability of insulin to exert its action, not just on carbohydrate metabolism but also on primarily in lipid metabolism, in addition to its anabolic and anti-catabolic actions [4,5].

Physical exercise has been widely used to protect the body against the decreased responses to the biological actions of insulin in peripheral tissues and to reduce the metabolic changes that are produced by insulin resistance. Physical exercise leads to increased glucose uptake [6,7], particularly in muscle and adipose tissue, by promoting the translocation of Glut-4 [8,9] to the membrane during muscle contraction. Exercise also causes a reduction of body fat, increased oxidation of adipose tissue [10], decreased activity of inflammatory proteins that have a negative effect on insulin action, an improvement in lipid profiles, and increased sensitivity to insulin [8].

Therefore, physical training plays an important role in modulating the metabolic response that is caused by insulin resistance in T2DM [11,12]. However, there is a lack of direct evidence regarding the preventive effect of exercise on the corporal issues that comprise T2DM, as such T2DM studies of humans are difficult to realize due to the required intensity of effort and schedule and training protocols. Therefore, animal models provide the most suitable means of studying this disease. Thus, chemically induced diabetes in animals has been widely used as an experimental model for studying complications caused by diabetes [13] and the effects of physical exercise on insulin resistance and fat metabolism.

Oliveira et al. [14], using a neonatal alloxan model, found higher levels of FFA and liver lipids in alloxan animals. These values were reduced after 8 weeks of continuous swim training at moderate intensity, which indicates the importance of physical exercise on fat metabolism. Ribeiro et al. [15] found no change in the FFA content of animals when alloxan was administered in rats up to 6 days old. For streptozotocin-induced diabetes in adult rats, however, the authors found higher FFA concentrations in animals that underwent high-intensity acute exercise [16]. After analyzing the lipid metabolism of alloxan adult animals, Moura et al. [17] found no difference in the concentrations of triglycerides, total cholesterol, and FFAs in animals after 44 days of continuous training with moderate intensity swimming compared to controls. Barakat et al. [18] showed that the ability of the livers of alloxan animals to synthesize total lipids, diglycerides, and triglycerides was similar to that of control animals. After 7 days of moderate intensity training on a treadmill, the same result was found. Alloxan-induced diabetes resulted in hypertriglyceridemia in adult animals [19]. Continuous swim exercise at moderate intensity reduced triglyceride levels [19]. The authors have suggested that this change in the triglyceridemia of animals induced by physical training is due to a modulation of lipoprotein lipase activity [20,21].

There is little information available on the effects of neonatal alloxan administration and the application of different physical exercise protocols on lipid metabolism. Therefore, this study aimed to compare the effects of continuous, intermittent, and strength training on serum and tissue variables on the lipid metabolism of alloxan rats.

## Materials and methods

### Animals

Male and female adult Wistar rats (90 days) were used in the study. The Central Animal Laboratory of the Paulista State University (UNESP), Botucatu provided the animals. The rats were housed in the vivarium of the Biodynamic sector of the Department of Physical Education of UNESP, Rio Claro, which was kept at room temperature (25 ± 1°C) with a 12/12 hour light/dark photoperiod with lights on from 6:00 to 18:00. The rats were provided free access to water and food (balanced diet for rodents, Purina®). The Ethics Committee for Animal Experimentation of the University of Taubaté (Comitê de ética para experimentação animal / Universidade de Taubaté, CEEA/UNITAU) approved the procedures that were adopted for the animals under protocol CEEA/UNITAU No. 019/08.

### Neonatal application of alloxan

At six days of age, male offspring with an average weight of 11.9 ± 1.2 g were administered intraperitoneal injections (250 mg/kg body weight) of alloxan monohydrate that was dissolved in 0.01 M citrate buffer, pH 4.5 [22], after fasting for 15 hours. Rats of the same age and gender injected with the vehicle (citrate buffer) were used as controls. The neonates were then distributed such that each mother breastfed eight pups.

### Design and experimental groups

**Figure 1:**
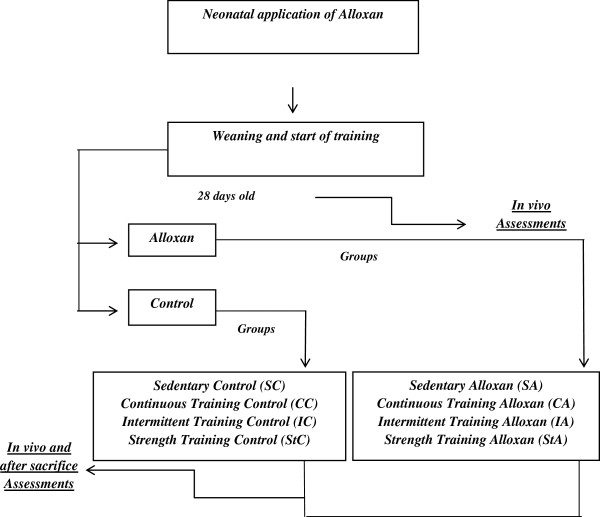
Experimental design.

At 28 days of age, the offspring comprised 8 groups of 10 animals each, which were observed until 120 days of age (Figure 1).

– Sedentary control (SC): rats injected with citrate buffer that did not subjected training.

– Continuous training control (CC): rats injected with citrate buffer that were subjected to the continuous training program.

– Intermittent training control (IC): rats injected with citrate buffer that were subjected to the intermittent training program.

– Strength training control (StC): rats injected with citrate buffer that were subjected to the strength training program.

– Sedentary alloxan (SA): rats injected with alloxan that did not subjected training.

– Continuous training alloxan (CA): rats injected with alloxan that were subjected to the continuous training program.

– Intermittent training alloxan (IA): rats injected with alloxan that were subjected to the intermittent training program.

– Strength training alloxan (StA): rats injected with alloxan that were subjected to the strength training program.

### Exercise training

The animals underwent an initial period of adaptation. First, they were allowed to acclimate to the aquatic environment for 15 minutes. On the following day, the level of water was increased, and the rats were allowed to swim for 15 minutes. On the 3^rd^ day the water level was adjusted to the level at which the rats trained, and they were allowed to swim for 25 minutes. On the 4^th^ day, they swam for 30 minutes with a load attached to the back that was equivalent to 3% of their body weight. On the 5^th^ day, the duration of the session was increased to 40 minutes with a load equivalent to 5% of their body weight. Following this adaptation period, the training period was started.

All of the exercise programs were begun upon weaning of the pups and were continued throughout 120 days of study. For the continuous exercise program, animals swam for 1 hour (uninterrupted) each day, 5 days per week, in individual tanks (50 cm high x 25 wide) while supporting a load of 5% of their body weight. This intensity of effort corresponded to the metabolic aerobic / anaerobic transition for the rats while swimming [23]. For the intermittent program, animals alternated between 30 s of swimming activity and 30 s of rest in individual tanks (50 cm high x 25 wide) for a total of 20 minutes per day, 5 days per week, while supporting a load of 15% of their body weight (adapted from Braga et al. [24] protocol). The total weekly training loads (TWT) of the training protocols were equal. According to Araújo et al. [25], the TWT corresponds to the sum of the training stimuli, which is obtained by taking the product of exercise time (t) and intensity (%). Thus, in the present study, the continuous training had a TWT of 60 min x 5% = 300%, which is equivalent to the intermittent training TWT of 20 min x 15% = 300.

The animals that underwent strength training were subjected to four series of 10 jumps in individual tanks of water alternated with 1 min of rest between each set, five days per week, while supporting a load of 50% of their body weight [26]. The water temperature was maintained at 31°C ± 1°C during the exercise, as this temperature is considered to be thermally neutral in relation to the temperature of rats [27,28].

### *In vivo* assessments

#### Insulin sensitivity

To estimate insulin sensitivity an insulin tolerance test (ITT) was performed at 28 days. The test consisted of an insulin solution (30 mU/100 g of body weight) that was administered subcutaneously in the dorsal region. Blood samples (25 μL) for glucose determination were collected in heparinized capillary tubes via a small incision in the end of the tail after 0, 30, 60, and 120 minutes (Kitt Glucose, Laborlab: CAT nº 02200, Guarulhos, SP, Brazil). The single tail incision was sufficient for obtaining all of the samples. The disappearance rate of glucose (Kitt) expressed in %/minute was calculated using the formula (0.693/t/2) X100. Blood glucose level (t/2) was calculated using the curve of the least square analysis of the serum glucose levels. The analysis revealed a linear decrease in blood glucose following insulin administration [29].

#### Stress test

Stress tests were performed to evaluate the effect of training, and our analysis was focused on blood lactate kinetics at 120 days of age. The rats trained in the continuous protocol swam uninterrupted for 30 min while supporting a load of 5% of their body weight. The animals trained in the intermittent protocol were subjected to 30 s of swimming alternated with 30 s of rest, totaling 20 min, while supporting a load with 15% of their body weight. Finally, the rats trained in the strength protocol underwent four series of 10 jumps in the water with 1 min of resting between each set while supporting a load of 50% of their body weight. For comparison, the sedentary rats were also subjected to the tests described above. Blood samples (25 μL) were collected in heparinized capillary tubes to analyze lactate concentrations. Blood was collected at rest, at every 5 minutes of exercise for the continuous protocol, and at every 5 min of effort in the intermittent protocol. For the strength protocol, collections were made after the completion of each series of jumps and at 5, 7, 9, 13, and 15 minutes after the end of the four series. Blood was collected via a small cut at the distal end of the tail. A single incision at the beginning of the test was sufficient for obtaining all of the samples. Lactate levels were obtained using the enzymatic method [30].

### Sacrifice of animals

At 120 days of age, to obtain the biological material, the animals were sacrificed via decapitation after deeply inducing anesthesia with amobarbital sodium (15 mg/kg body weight) without prior fasting 48 hours after the oral glucose tolerance tests and/or 48 hours after the last training session.

#### Assessments after sacrifice

**Blood** Blood samples were collected to verify the concentrations of free fatty acids (FFAs), triglycerides, total cholesterol, and total lipids using a commercial spectrophotometric kit (Laborlab®, Guarulhos, SP, Brazil).

#### Tissue

**Adipose tissue** The adipose tissue of the posterior, mesenteric, and retroperitoneal subcutaneous regions was removed and weighed for the determination of total lipids. Excisions of the various fat deposits were performed according to [31]. The concentrations of lipids in the deposits were determined using the procedure described by [32].

##### Liver, heart, and gastrocnemius muscle

The concentrations of total lipids were evaluated in the liver, heart, and gastrocnemius.

### Statistical analysis

The data analysis was performed using Student’s-t test or a two-way analysis of variance ANOVA followed by the Newman-Keuls post hoc test, where appropriate. In all cases, the levels of significance were preset at 5% (p<0.05).

## Results

**Figure 2:**
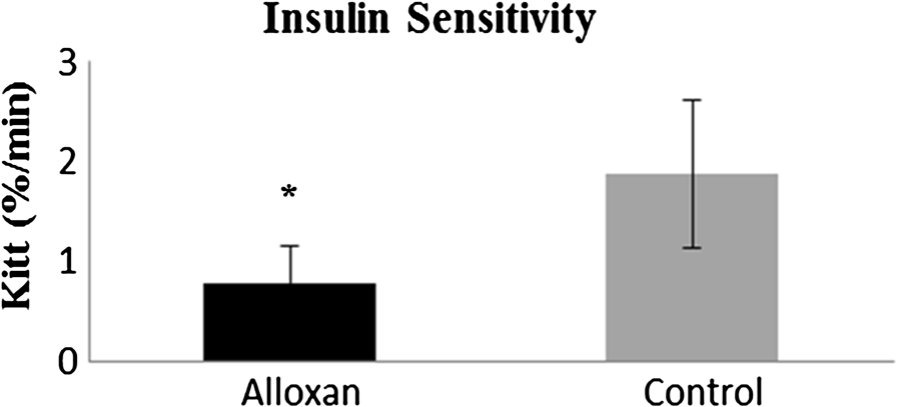
**Serum glucose disappearance rate (Kitt, %/min) during the insulin tolerance test (ITT) at weaning (28 days).** The results are expressed as the mean ± SD, n = 10 animals per group. The symbol * indicates significant difference (Student’s t test, p < 0.05) between groups (alloxan and control).

At 28 days of age, an insulin tolerance test (ITT) was performed to evaluate insulin sensitivity, which was evaluated using the Kitt glucose disappearance rate (%/min). Alloxan animals exhibited lower disappearance rates compared to controls, which shows the effectiveness of the neonatal administration of alloxan for the installation of clinical picture of insulin resistance (Figure 2).

**Figure 3:**
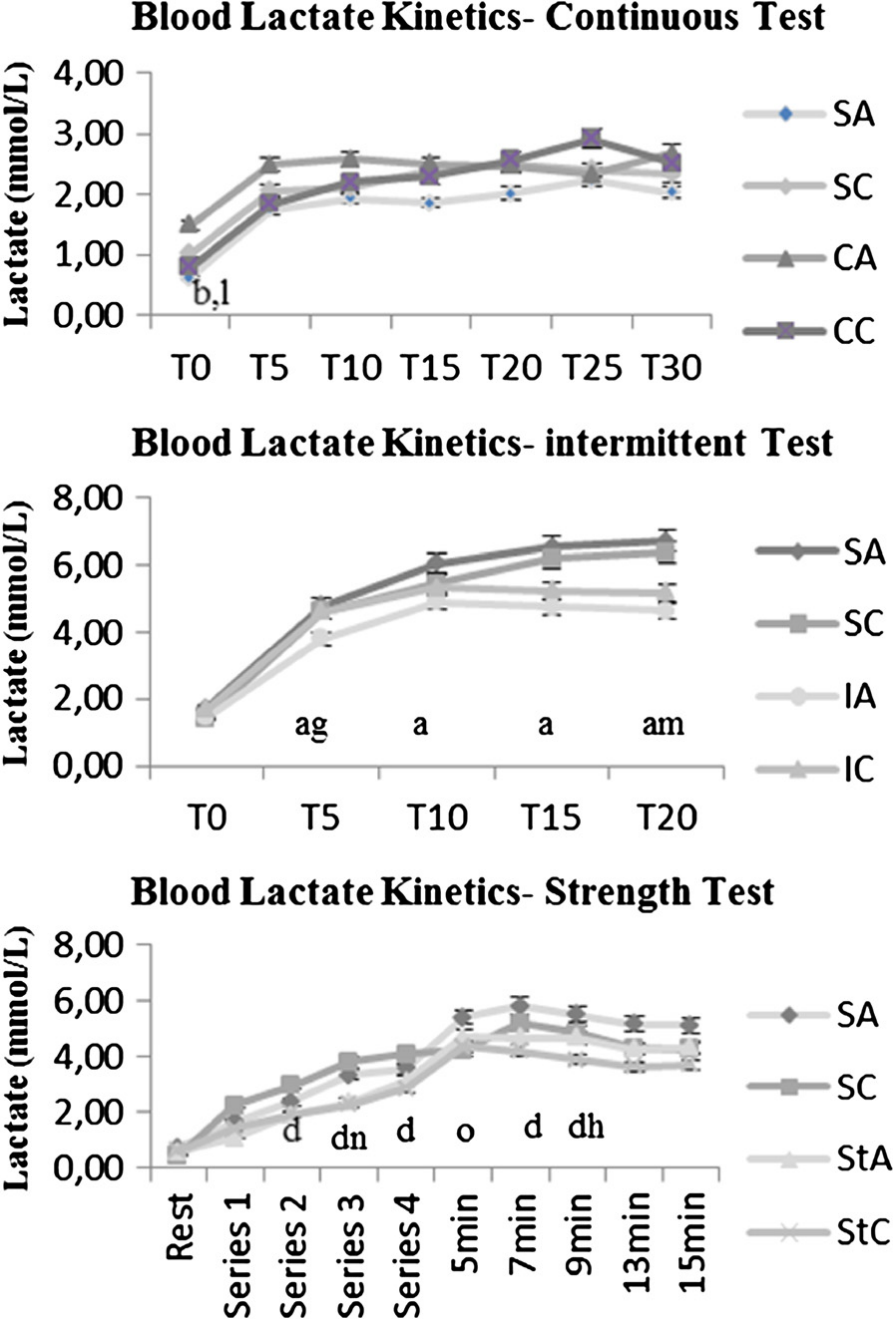
**Blood lactate kinetics during the stress test for the continuous, intermittent, and strength training groups.** Analyses were conducted at the end of the experiment. The results are expressed as the mean ± SD, n = 10 animals per group. SA: sedentary alloxan, SC: sedentary control, CA: continuous training alloxan, CC: continuous training control, IA: intermittent training alloxan, IC: intermittent training control, StA: strength training alloxan and StC: strength training control. The letters indicate significant differences (two-way ANOVA (3x2) and Newman-Keuls post hoc, p < 0.05)) among the groups. a = significant difference between SA and IA; d = significant difference between SC and StC; g = significant difference between IA and IC; h = significant difference between StA and StC; m = significant difference between SC and IC; n = significant difference between AS and StA, o = significant difference between SA and SC.

In addition, stress tests were performed at 120 days of age to evaluate the effect of the different training protocols, continuous, intermittent, and strength, through lactate kinetics (Figure 3). The analysis of the lactate concentrations for the continuously trained alloxan group (CA) revealed higher lactacidemia at rest compared to the corresponding sedentary (SA) and trained (CC) control groups. Differences in the test were also found regarding the intermittent training. According to the lactate kinetics, the sedentary alloxan group (SA) showed higher values than the trained alloxan group (IA), which in turn showed lower concentrations than the corresponding control group (IC) at rest. Furthermore, the sedentary control group (SC) showed lower lactate concentrations at the end of the test compared to the trained control (IC). Lactacidemia analysis of the animals submitted to the strength training protocol revealed that the sedentary control animals (SC) had higher concentrations throughout the test than the trained control animals (StC). The sedentary alloxan animals (SA) showed higher values than the sedentary control (SC) and alloxan trained (StA) groups 5 min after termination of the test and after the third series of jumps, respectively. Nine minutes after the test, the trained alloxan animals (StA) showed higher concentrations than the corresponding control group (StC), and increased lactacidemia was found in the alloxan and sedentary animals.

FFA, triglyceride, total cholesterol, and total serum lipids levels are shown in Table 1. Regarding FFA values, the sedentary alloxan animals (SA) showed higher values than the alloxan animals after continuous training and intermittent swimming (CA and IA). The control group after continuous training (CC) had lower FFA values than the sedentary control (SC), intermittent training (IC), and strength (StC) groups. Moreover, alloxan animals that underwent intermittent (IA) and strength (StA) training showed lower values of FFAs compared to the corresponding controls (IC and StC). No differences were found among the triglyceride concentrations. The continuous training protocol was less effective than the strength training protocol in reducing cholesterol levels in alloxan animals (CA > StA). Total serum lipid values also showed that intermittent training increased serum levels in alloxan animals (IA > SA, CA, StA and IC). With regard to the lipids assessed in the retroperitoneal, mesenteric, and subcutaneous portions of the animals adipose tissue, no differences were found (Table 2).

**Table 1:** FFA, triglyceride, total cholesterol, and serum lipid levels measured at 120 days of age

**Variables**	**SA**	**SC**	**CA**	**CC**	**IA**	**IC**	**StA**	**StC**
**Serum FFA (μEq / L)**	539.2 ±	498.6 ±	392.5 ±	334.9 ±	390.0 ±	564.8 ±	438.2 ±	659.1 ±
	116.5_ab_	111.7_cd_	111.3	105.6_ef_	92.4 _g_	120.7	94.4 h	78.7
**Triglycerides (mg / dL)**	215.3 ±	245.0 ±	274.0 ±	197.5 ±	278.3 ±	262.7 ±	228.5 ±	280.3 ±
	47.8	69.2	67.1	57.5	93.9	58.2	51.8	106.1
**Total cholesterol (mg / dL)**	85.4 ±	89.1 ±	88.5 ±	85.9 ±	84.9 ±	77.0 ±	70.4 ±	79.9 ±
	14.8	14.2	21.1 _i_	15.3	9.1	6.7	7.1	10.6
**Total lipids (mg / dL)**	345.5 ±	328.0 ±	379.7 ±	315.9 ±	444.1±	362.9 ±	330.1 ±	329.1 ±
	53.3 _a_	73.6	58.3_j_	53.6	107.7 _g k_	54.7	53.3	78.8

The results expressed as the mean ± SD, n = 10 animals per group. SA: sedentary alloxan, SC: sedentary control, CA: alloxan continuous training, DC: continuous training control, IA: alloxan intermittent training, IC: intermittent training control, StA: alloxan strength training and StC: strength training control. The letters indicate significant differences (two-way ANOVA 3x2) and Newman-Keuls post hoc, p < 0.05)) among the groups. a = significant difference between SA and IA; b= significant difference between SA and CA; c = significant difference between SC and CC; d = significant difference between SC and StC; e = significant difference between CC and IC; f = significant difference between CC and StC, g = significant difference between IA and IC; h =significant difference between StA and StC; i = significant difference between CA and StA; j = significant difference between CA and IA; k = significant difference between IA and StA.

**Table 2:** Adipose tissue lipids assessed in the retroperitoneal, mesenteric, and subcutaneous portions of the animals at 120 days

**Lipids Adipose Tissue**	**SA**	**SC**	**CA**	**CC**	**IA**	**IC**	**StA**	**StC**
**Retroperitoneal (Mg/100 mg)**	62.6 ±	54.2 ±	57.0 ±	62.5 ±	53.2 ±	49.5±	60.1 ±	55.3 ±
	−11.7	0.1515.0	17.8	9.7	8.6	7.5	16.0	16.0
**Mesenteric (Mg/100 mg)**	44.5 ±	43.6±	43.6±	36.7 ±	42.0 ±	38.0 ±	36.7 ±	42.5 ±
	11.4	11.4	10.2	10.5	7.4	3.2	4.3.	8.7
**Subcutaneous (Mg/100 mg)**	43.2±	43.0±	41.1±	40.7±	37.8±	42.8±	38.9±	32.5±
	8.0	9.4	8.0	12.0	6.8	6.0	6.7	4.2

The results are expressed as the mean ± SD, n = 10 animals per group. SA: sedentary alloxan, SC: sedentary control, CA: alloxan continuous training, CC: continuous training control, IA: alloxan intermittent training, IC: intermittent training control, StA: alloxan strength training and StC: strength training control. The letters indicate significant differences (two-way ANOVA (3x2) and Newman-Keuls post hoc, p <0.05)) among the groups.

Table 3 summarizes the results relating to gastrocnemius muscle, heart, and liver tissue lipids that were measured in the animals at 120 days of age. Differences were found only in the liver; the alloxan animals that were subjected to continuous swim training showed lower values (CA <SA, IA, StA, and CC), which demonstrates the importance of aerobic training for controlling possible lipid changes caused by insulin resistance in liver tissue.

**Table 3:** Gastrocnemius muscle, heart, and liver tissue lipids measured in animals at 120 days of age

**Lipids**	**SA**	**SC**	**CA**	**CC**	**IA**	**IC**	**StA**	**StC**
**Gastrocnemius muscle (Mg/100 mg)**	3.0 ±	2.6±	2.9±	2.4 ±	2.6±	2.6±	2.7±	2.4±
	0.8	0.5	0.8	0.4	0.5	0.8	0.4	0.5
**Liver (Mg/100 mg)**	5.7 ±	5.0±	4.5 ±	5.8 ±	5.9 ±	5.5 ±	5.7 ±	5.4 ±
	1.0_b_	0.9	0.9_i j l_	0.9	0.9	0.6	0.7	0.6
**Heart (Mg/100 mg)**	5.2±	5.5±	7.2±	6.2±	6.3±	6.3±	6.5±	6.2±
	0.8	1.4	2.1	1.1	6.3±	1.6z	1.9	0.9

The results are expressed as the mean ± SD, n = 10 animals per group. AS: sedentary alloxan, SC: sedentary control, CA: alloxan continuous training, CC: continuous training control, IA: alloxan intermittent training, IC: intermittent training control, StA: Alloxan strength training and StC: Strength training control. The letters indicate significant differences ((two-way ANOVA (3x2) and post hoc Newman-Keuls, p <0.05)) among the groups. b = significant difference between AS vs. CA; i = significant difference between CA vs. StA; j = significant difference between CA vs. IA; l = significant difference between CA vs. CC.

## Discussion

Type 2 Diabetes mellitus (T2DM) is a chronic disease that affects approximately 90-95% of diabetics [5] and has grown considerably in recent years. The cause of T2DM is a combination of a resistance to insulin action as well as compensatory responses of hormone secretion. This combination results in alterations in the metabolism of carbohydrates, lipids, and proteins due to a deficient action of insulin in peripheral target tissues, which have a decreased response at one or more points of the hormone signaling pathway [4,5]. The risk of developing type 2 diabetes increases with age, obesity, and physical inactivity, all of which contribute to a metabolism imbalance, principally in lipid profiles. The present study aimed to compare the effects of continuous, intermittent, and strength training on serum and tissue variables on the lipid metabolism of alloxan rats.

The present study data showed an efficacy of neonatal alloxan administration, proved by the changes in insulin sensitivity in alloxan animals when assessing the serum glucose disappearance rate in ITT after 28 days of age. These findings are consistent with previous studies conducted using a neonatal alloxan model [12,33,34], which showed lower insulin sensitivity in alloxan animals.

Chronic physical training has an important role in improving insulin sensitivity [35,36] and the lipid profile [8] in T2DM, but the optimal level of effort that should be applied remains unknown. The present study used three physical training protocols with different intensities of effort to evaluate the effects of aerobic and anaerobic capacity on alloxan animals.

Assessments of aerobic capacity are performed using procedures that investigate a transition zone during exercise to ascertain where there is a predominance of aerobic metabolism over anaerobic metabolism in the supply of adenosine triphosphate to muscle activity. The procedures utilize an analysis of blood lactate concentration as a metabolic parameter because it is a reliable measure of the transition for characterizing the chronic effects of training [37] as well as the effort involved. In the present study, a stress test was conducted to measure lactacidemia in animals after 12 weeks of continuous, intermittent, and strength swimming.

No differences were found in lactate kinetics among the groups that underwent the continuous training protocol. These findings are consistent with Ribeiro et al. [38], who conducted a study using a neonatal alloxan model that demonstrated similar lactacidemia among groups. The response of sedentary animals to the test could be interpreted as moderate intensity with a predominance of aerobic metabolism, given the values of lactate that were achieved and the moderate intensity at the start of the training program. For the intermittent training protocol, although there was the same total weekly load as in the continuous training protocol, a lower lactate disappearance rate in sedentary animals was found, which showed that training had an effect. By measuring the lactate kinetics related to strength training, a higher degree of lactacidemia in sedentary animals compared to controls was found throughout the test, which demonstrates the positive effect of the applied training. These results confirm the chronic effect of exercise in addition to demonstrating a positive response of alloxan animals to different protocols and intensities of effort.

Due to the relationship between changes in insulin sensitivity and lipid metabolism in T2DM and the role of physical exercise, the present study also assessed the animals lipid profile.

High levels of circulating FFAs are correlated with lower phosphorylation and insulin activation (IRS/PI3q) [39]. The presence of FFAs provides a direct relationship with insulin resistance that may result from the accumulation of triglycerides in muscle and liver. Furthermore, the increase of these metabolites due to the oxidation of fat in muscle is capable activating protein kinase C (PKC) and inducing serine phosphorylation of the insulin receptor (IR) and its substrates, which are important mechanisms that explain the relationship between the accumulation of fat tissue and resistance to insulin [39,40]. The present study thus assessed the serum concentrations of FFAs, triglycerides, total cholesterol, and total lipids in addition to lipid levels in adipose tissue, the gastrocnemius muscle, the liver, and the heart.

The serum concentration of FFAs showed a higher concentration in sedentary alloxan animals, but a reduction in the level was found after continuous and intermittent swim training. Additionally, intermittent and strength training were more effective in reducing the level of fatty acids in alloxan animals than in controls. Oliveira et al. [14] found similar results in a neonatal alloxan model. Alloxan animals showed higher levels of FFAs that diminished after continuous swimming. Stolen et al. [41] found results in db/db animals that were similar to the FFA concentrations after 13 weeks of intermittent training. Ghelfi et al. [42] found no differences in the concentrations of FFAs in type 1 diabetic subjects after intermittent training of high and moderate intensity. Similar results were found in the serum concentrations of FFAs in alloxan-induced diabetic adult animals [17]. Holten et al. [43] found similar results in a study of type 2 diabetic subjects after strength training. Thus, it is noted that the training protocols applied in the current study were effective in reducing the accumulation of serum FFAs in the animals. Therefore, they likely play an important role in the prevention and treatment of metabolic disturbances in lipid metabolism that are caused by insulin resistance in T2DM. No change was observed between the groups in the concentration of triglycerides. This result differs from the study of alloxan adult animals [18], which reported the animals having lower concentrations of triglycerides after 7 days of training on a treadmill. In the present study, strength training was more effective in reducing total cholesterol levels in alloxan animals, and strength and continuous training were better for the maintenance of total serum lipids. In an experimental model of alloxan-induced diabetes in adult animals, Moura et al. [17] found no significant differences in serum cholesterol levels even after 44 days of continuous training at moderate swimming intensity. In a study using Zucker Diabetic Fatty (ZDF) rats as a T2DM model, diabetic animals had reduced serum cholesterol levels after 12 weeks of swim training at moderate intensity, suggesting the importance of physical training on the lipid profile of T2DM [44].

Many individuals with T2DM are overweight or obese. Thus, their distribution [45] and accumulation of lipids in adipose tissue differ. Therefore, analyses of lipid levels in the adipose tissue in the retroperitoneal, mesenteric, and subcutaneous regions of rats were performed, although no differences were found in the concentrations of these lipids.

Obesity and alterations in the lipids metabolism has been shown as a cause of insulin resistance [46,47]. In recent years studies have shown a relationship between organ dysfunction and lipids storage in tissues such as liver, heart, pancreas, and skeletal muscle [48]. It is known that exercise training can reduce the changes in lipid metabolism of high-risk subjects for development of T2DM [49].

Therefore, the present study also evaluated total lipid levels in the gastrocnemius muscle, liver, and heart. Differences were noted only in liver tissue for alloxan animals that underwent continuous training, which showed lower concentrations. These results corroborate with those of Oliveira et al. [14] who, using the same neonatal alloxan model, reported a higher use of lipids in the liver after physical training, thereby reducing the amount in the tissue. Moura et al. [17] found similar data in a type 1 diabetes experimental model, which highlights the importance of exercise in the control of total lipids in liver tissue.

In summary, physical training at different intensities of effort and on different schedules was key in the reduction of lipid levels and control of changes in lipid metabolism in alloxan animals. It may therefore be concluded that both high and moderate intensity training were effective in improving the lipid profile of alloxan animals. Therefore, exercise can play an important role in the prevention and treatment of metabolic alterations in lipid metabolism that are caused by insulin resistance T2DM.

## Competing interests

The authors declare that they have no competing interests.

## Authors’ contributions

CR conceived the study, developed the study protocol, reviewed the references, collected and analyzed the data, and wrote the paper. LTC, RAD, MBA, JDB and ACSS , participated in the design of the study, reviewed the manuscript, collected the data, and collaborated on the biochemical dosages. MARM conceived the study, participated in its design and coordination and helped in the drafting of the manuscript. All authors read and approved the submission of the final manuscript.
